# Steroid‐Induced Mental Disorders in Oncology Patients: A 10‐Year Retrospective Case Series Review

**DOI:** 10.1002/pon.70137

**Published:** 2025-04-19

**Authors:** Niall Seymour, Muhammad Fahmi Ismail, Kieran Doherty, Ann Bowler, Richard Bambury, Shahid Iqbal, Eugene M. Cassidy

**Affiliations:** ^1^ University College Cork Cork Ireland; ^2^ Cork University Hospital Cork Ireland; ^3^ St Vincent's University Hospital Dublin Ireland

**Keywords:** cancer, case series review, corticosteroid, Ireland, oncology, psycho‐oncology, steroid‐induced mental disorder, steroid‐induced psychosis

## Abstract

**Objective:**

Patients with cancer are commonly prescribed corticosteroids for a variety of indications. Corticosteroids have long been known to affect mental state. Neuropsychiatric effects range from insomnia, cognitive impairment, and mood symptoms to psychosis and mania. In this study, we aimed to investigate the demographics, steroid exposure, referring indications, symptom profiles, and subsequent treatments of steroid‐induced mental disorders in oncology patients.

**Methods:**

We conducted a retrospective chart review of patients diagnosed with a steroid‐induced mental disorder, as assessed by the psycho‐oncology team in Cork University Hospital from 2626 referrals to the service between January 2010 to December 2019.

**Results:**

In total, 297 patients had a diagnosis of steroid‐induced mental disorder (11% of referrals). 60.6% were female and mean age ± standard deviation (SD) was 57.5 ± 12.9 years. Breast cancer was the most frequent malignancy among females. Haematological cancer was the most frequent among males and the second most frequent among females. The most commonly prescribed steroid was dexamethasone, followed by prednisolone. The median (interquartile range [IQR]) cumulative weekly prednisolone equivalent dose was 186 mg (125–350 mg), with a median (IQR) duration of steroid exposure before symptom onset of 14 (6–47) days. The most frequently recorded symptoms following psychiatric assessment included insomnia, anxiety, and irritability. Psychotropics were commenced in *n* = 174 (74%) patients, with antipsychotics prescribed to 62.1%. A watchful wait approach was adopted for 25.5% of the patients. 90.2% (*n* = 185/205) of the patients experienced either complete or partial resolution of the symptoms at their first clinical review.

**Conclusions:**

Steroid‐induced mental disorders can cause significant comorbidity in patients receiving cancer treatment. The most common symptoms recorded during assessment included insomnia, anxiety, and irritability.

## 1 Background

### 1.1 Use of Systemic Corticosteroids in Oncology

Widespread adoption of systemic corticosteroid therapy has been a defining trend in cancer care over the past several decades. Corticosteroids are particularly effective in treating spinal cord compression, superior vena cava syndrome, brain metastases, and bowel obstruction [1]. Despite limited scientific evidence of their efficacy, corticosteroids are also often prescribed to patients with cancer as adjuvant analgesics, and appetite stimulants [2, 3].

However, corticosteroids have notable physical and neuropsychiatric side effects that impact patient outcomes. Understanding these effects and associated risks is crucial for prescribing physicians. Physical side effects of corticosteroids are wide‐ranging, including musculoskeletal issues; metabolic disturbances like cushingoid features, adrenal suppression, and hyperglycaemia; cardiovascular complications; gastrointestinal disturbances; increased infection risk; dermatological issues; and eye conditions such as cataracts and glaucoma [4].

### 1.2 Steroid‐Induced Mental Disorders

The use of systemic corticosteroids has been associated with neuropsychiatric side effects, including affective, behavioural, and cognitive manifestations [5]. A steroid‐induced mental disorder, therefore, is a substance‐induced mental disorder that can represent a spectrum of symptoms ranging from mild changes in mood and sleep disturbances to suicidal ideation and overt psychosis [6].

Various theories regarding the pathophysiology of neuropsychiatric side effects of corticosteroids have been proposed, such as steroid interference with the cortical pathway of the hypothalamic‐pituitary‐adrenal axis, impact on monoamine levels, changes in the dopaminergic and cholinergic pathways, and adverse effects on hippocampal neurons [7, 8, 9, 10, 11]. These side effects can be attributed to several reasons, and their underlying mechanisms may differ among patients and doses. Generally, the incidence of side effects of systemic corticosteroids, both physical and neuropsychiatric, increases with longer durations and higher doses [12, 13]. Of note, excess cortisol has long been implicated in the pathogenesis of mood disorders. Various studies have reported evidence of clinically significant mood disturbance in more than 50% of patients with Cushing's syndrome, an umbrella term describing the clinical manifestations of the overproduction of cortisol [14, 15, 16]. Whether this mood disorder is a direct biochemical result of systemic cortisol excess is unclear. However, both pharmacological reduction of cortisol levels and the inhibition of cortisol function by glucocorticoid receptor blockade have been reported to ameliorate mood in people with Cushing's syndrome, as well as in otherwise healthy depressed subjects [17, 18].

The treatment of steroid‐induced mental disorders is highly variable because evidence to support a formal management approach for affected patients or psychiatric presentations is limited. Traditionally, affected patients are managed by tapering and discontinuing corticosteroids, with first‐generation antipsychotics often used in severe cases [19]. Some evidence in the literature supports other pharmacological treatments, including second‐generation antipsychotics (SGAs), lithium, selective serotonin reuptake inhibitors, tricyclic antidepressants, and mood stabilisers [19]. However, most published literature is limited to case series and reports. Furthermore, the term ‘steroid‐induced psychosis’ remains a commonly used term in the literature despite being outdated, and is often actually used to refer to a heterogeneous combination of neuropsychiatric effects that commonly do not involve psychosis [6]. This makes identifying appropriate treatment strategies difficult because psychotropic regimens often are not tailored to the specific symptoms that patients with steroid‐induced mental disorders experience.

### 1.3 Knowledge Gap

A previous systematic review on steroid‐induced mental disorders in patients with cancer highlighted a pronounced deficiency in data regarding prevention and treatment strategies for this group, possibly owing to limitations in research methodologies and a lack of large‐scale studies, patient diversity, and application challenges in clinical settings [7]. The review emphasised how neuropsychiatric side effects can disrupt cancer treatments and impact on outcomes [7]. Given the baseline heightened suicide risk among patients with cancer, the potential side effects of corticosteroids introduce substantial additional risks, necessitating careful consideration by physicians [20, 21]. Ireland's National Cancer Strategy 2017–2026 proposes the expansion of specialist psycho‐oncology services and calls for high‐quality studies to enrich the intervention evidence base [22]. Accordingly, this study aims to address some of these gaps, providing insights that can inform both clinical practice and future research directions in the management of steroid‐induced mental disorders in oncology patients.

### 1.4 Study Objectives

- To assess the demographics, including medical history and steroid use, of patients with cancer diagnosed with steroid‐induced mental disorders.
- To illustrate the frequency and range of psychiatric symptoms experienced by these patients.
- To provide an overview of the treatments initiated by the psycho‐oncology team in the given cohort of patients.

## 2 Methods

This study was a retrospective chart review conducted at Cork University Hospital (CUH), one of the eight regional oncology centres in Ireland and the largest university teaching hospital in the country [23]. Given that only archived data was used in this study, the requirement for informed consent was waived. Ethical approval was granted by the Clinical Research Ethics Committee of Cork Teaching Hospitals (Approval No. ECM4(b)12/08/19, dated 23 February 2019).

### 2.1 Study Design and Participants

Patients with cancer who were diagnosed with steroid‐induced mental disorders following assessments by the psycho‐oncology team at CUH between January 2010 and December 2019 were included in this study. The psycho‐oncology team comprised a multidisciplinary liaison psychiatry service. The assessment was conducted jointly by clinical nurse specialists and a senior psychiatrist. The patients were identified using a database that recorded the demographic details and diagnoses from the assessments undertaken by the service. All patients included in this study were aged ≥ 18 years and were diagnosed with cancer.

### 2.2 Study Measures

The collected data included information regarding patient demographics, cancer diagnoses and staging, medical and psychiatric comorbidities, steroid use (including type, dosage, and duration of treatment), reasons for referral, symptom profiles, interventions by the psycho‐oncology team, and subsequent responses to treatment. Supporting Information S1: Appendix A presents the data collection sheets used in this study.

### 2.3 Data Analysis

The data were analysed in line with the study objectives. Descriptive statistics were used to summarise patient demographics, indications for referral, and clinical presentation. Statistical tests, including chi‐squared tests and *t*‐tests, were used to further analyse the data. Additionally, patients' responses to treatment were evaluated. Given the data and time constraints of this project, the evaluation was limited to each patient's clinical response at the time of their first clinical review after treatment initiation. The Strengthening the Reporting of Observational Studies in Epidemiology checklist was used to ensure that all aspects of this study were comprehensively reported [24].

## 3 Results

Data from 297 patients were included in this retrospective chart review, comprising 11% of the total referrals (*n* = 2626) to the service. As the data for all analysed variables were not available for all 297 patients, in some cases, the results were presented using an *n*/*N* format to highlight the number of patients who had a certain variable (*n*) out of the number of patients for whom data regarding the particular variable were available (*N*).

### 3.1 Patient Demographics

The demographic and clinical characteristics of the 297 patients included in this study are shown in Table 1. There was a significantly higher proportion of female patients in this cohort (60.6% *χ* ^2^ = 12.986, *p* < 0.001). Additionally, females (55.65 ± 12.8 years) in our sample were younger than males (60.03 ± 12.7 years; *t* = 2.862, *p* = 0.002).

**TABLE 1:** Patient demographics and clinical characteristics.

	Overall	Female	Male
	*N* = 297	*N* = 180	*N* = 117
Age, mean ± standard deviation (years)	57.5 ± 12.9	55.7 ± 12.8	60.0 ± 12.7
Marital status, *n* (%)	*N* = 287	*N* = 174	*N* = 113
Married	179 (62.4%)	101 (58.0%)	78 (69.0%)
Single	54 (18.8%)	30 (17.2%)	24 (21.2%)
Separated	34 (11.8%)	27 (15.5%)	7 (6.2%)
Widowed	20 (7.0%)	16 (9.2%)	4 (3.5%)
Highest educational level, *n* (%)	*N* = 268	*N* = 164	*N* = 104
Primary	5 (1.8%)	1 (0.6%)	4 (3.8%)
Lower secondary	105 (39.2%)	49 (29.9%)	56 (53.8%)
Upper secondary	78 (29.1%)	53 (32.3%)	25 (24.0%)
Third level (non‐degree)	35 (13.1%)	28 (17.1%)	7 (6.7%)
Third level (degree or higher)	45 (16.8%)	33 (20.1%)	12 (11.5%)
Employment status, *n* (%)	*N* = 288	*N* = 177	*N* = 111
Employed/self‐employed	111 (38.5%)	69 (39.0%)	42 (37.8%)
Retired	96 (33.3%)	44 (24.9%)	52 (46.9%)
Homemaker	47 (16.3%)	47 (26.6%)	0 (0.0%)
Unemployed	30 (10.4%)	14 (7.9%)	16 (14.4%)
Student	4 (1.4%)	3 (2.0%)	1 (0.9%)
Cancer diagnosis, *n* (%)	*N* = 295	*N* = 179	*N* = 116
Haematological	78 (26.4%)	36 (20.1%)	42 (36.2%)
Breast	73 (24.7%)	72 (40.2%)	1 (0.1%)
Lung/thoracic	50 (16.9%)	29 (16.2%)	21 (18.1%)
Central nervous system	42 (14.2%)	12 (6.7%)	30 (25.9%)
Gastrointestinal	18 (6.1%)	11 (6.1%)	7 (6%)
Genitourinary	15 (5.1%)	10 (5.6%)	5 (4.3%)
Skin	7 (2.4%)	4 (2.2%)	3 (2.6%)
Head and neck	7 (2.4%)	2 (1.1%)	5 (4.3%)
Other/unknown	5 (2%)	3 (1.6%)	2 (1.7%)
First cancer diagnosis, *n* (%)	*N* = 288	*N* = 174	*N* = 114
Yes	255 (88.2%)	161 (92.5%)	94 (82.5%)
No	33 (11.4%)	13 (7.5%)	20 (17.5%)
Staging, *n* (%)	*N* = 224	*N* = 144	*N* = 80
Localised disease only	101 (45%)	61 (42.3%)	40 (50%)
Local spread	36 (16.1%)	24 (16.7%)	12 (15%)
Distant metastasis	87 (38.9%)	59 (41%)	28 (35%)
Brain malignancy or brain metastases, *n* (%)	*N* = 287	*N* = 173	*N* = 114
Yes	91 (31.7%)	46 (26.6%)	45 (39.5%)
No	196 (68.3%)	127 (73.4%)	69 (60.5%)
Current cancer treatment on assessment *n* (%)	*N* = 285	*N* = 173	*N* = 112
Chemotherapy	175 (61.4%)	115 (66.4%)	60 (53.4%)
Radiotherapy	45 (15.8%)	29 (16.8%)	15 (13.4%)
Combined chemo/radiotherapy	39 (13.7%)	21 (12.1%)	18 (16.1%)
Pre/post‐surgical	12 (4.2%)	2 (1.2%)	10 (8.9%)
Other oncological treatment	7 (2.5%)	1 (0.6%)	6 (5.4%)
No current treatment	6 (2.1%)	4 (2.3%)	2 (1.8%)
Pre/post‐transplant	2 (0.7%)	1 (0.6%)	1 (< 1%)
Medical comorbidities, *n* (%)	*N* = 297	*N* = 180	*N* = 117
Cardiovascular disease	69 (23.2%)	36 (20.0%)	33 (28.2%)
Endocrine disease	29 (9.8%)	20 (11.1%)	9 (7.8%)
Respiratory disease	22 (7.4%)	14 (7.8%)	8 (6.8%)
Musculoskeletal disease	22 (7.4%)	10 (5.6%)	12 (10.3%)
Neurological disease	7 (2.4%)	4 (2.2%)	3 (2.6%)
Other physical comorbidity	47 (15.8%)	29 (16.1%)	18 (15.4%)
No recorded physical comorbidity	101 (34.0%)	67 (37.2%)	34 (29.1%)
Psychiatric comorbidities, *n* (%)	*N* = 297	*N* = 180	*N* = 117
Depressive disorder	57 (19.2%)	39 (21.7%)	18 (10.2%)
Anxiety disorder	25 (8.4%)	17 (9.4%)	8 (6.8%)
Psychotic disorder	11 (3.7%)	7 (3.9%)	4 (3.4%)
Substance use disorder	8 (2.7%)	5 (2.8%)	3 (2.6%)
Personality disorder	5 (1.6%)	4 (2.2%)	1 (0.9%)
Bipolar disorder	2 (0.6%)	1 (0.6%)	1 (0.9%)
Other psychiatric comorbidity	9 (3.0%)	5 (2.8%)	4 (3.4%)
No recorded psychiatric comorbidity	180 (60.6%)	102 (56.7%)	78 (66.7%)

Breast cancer was the most frequently diagnosed cancer among females in the cohort, accounting for over 40% of all diagnoses. Haematological cancer was the most common diagnosis among men. Additionally, a significant proportion of the study population, 31% (*n* = 91) had oncological brain involvement, and 61% (*n* = 175) were receiving chemotherapy when they were referred to a psycho‐oncology service. Of the 297 patients included in this study, 68% (*n* = 201) had at least one medical and/or psychiatric comorbidity. Cardiovascular disease was the most common medical comorbidity, observed in 23.2% of the cohort. Depressive disorders were the most common psychiatric comorbidity, reported in 19.2% of the cohort.

### 3.2 Steroid Use

Dexamethasone was the most commonly prescribed steroid (84.5%, *n* = 251/297), followed by prednisolone (13.1%, *n* = 39/297). Steroids were administered orally in 79.9% (*n* = 227/284) of patients, intravenously (IV) in 3.5% (*n* = 10/284), and orally + IV in 16.5% (*n* = 47/284) of the patients. The median duration of steroid exposure before the onset of symptoms of a steroid‐induced mental disorder became apparent was 14 days (range, 1–746 days), with 50.4% (*n* = 123/244) of patients displaying symptoms within this timeframe. The weekly dose of steroids was converted to a prednisolone equivalent dose using standard glucocorticoid conversion charts. For patients receiving pulse or continuous steroid regimens, the average weekly dose was calculated by dividing the total dose by the treatment duration. The median cumulative weekly prednisolone equivalent dose for the cohort was 186 mg (range, 2–1280 mg), with 51.5% (*n* = 134/260) of patients prescribed ≥ 186 mg of weekly prednisolone equivalent dose. In total, 9.2% (*n* = 27/293) of the patients in this cohort had experienced a previous episode of a steroid‐induced mental disorder. An overview of these results, including a breakdown of the two most commonly used steroids, dexamethasone and prednisolone, is presented in Table 2.

**TABLE 2:** Steroid use.

Prescribed steroid, *n* (%)	*N* = 297
Dexamethasone	251 (84.%)
Prednisolone	39 (13.1%)
Hydrocortisone	3 (1.%)
Prednisone	2 (0.7%)
Other	2 (0.7%)

	Dexamethasone	Prednisolone	Overall
Route of administration, *n* (%)	*N* = 241	*N* = 36	*N* = 284
Oral	186 (77.2%)	36 (100%)	227 (79.9%)
IV	8 (3.3%)	0 (0%)	10 (3.5%)
Mixed oral/IV	47 (19.5%)	0 (0%)	47 (16.6%)
Cumulative weekly prednisolone equivalent dose, median (IQR)	175 (125–320) mg	400 (210–500) mg	186 (125–350) mg
Duration of steroid exposure before onset of symptoms of a steroid induced mental disorder, median (IQR)	14.5 (7–42) days	14 (5–76) days	14 (6–47) days
Previous episode of a steroid‐induced mental disorder, *n* (%)	*N* = 248	*N* = 37	*N* = 292
Yes	17 (6.9%)	8 (21.6%)	27 (9.2%)
No	231 (93.1%)	29 (78.4%)	265 (90.8%)

### 3.3 Pre‐Assessment of Psychotropics

Notably, 57.6% (*n* = 171/297) of the cohort were already prescribed at least one psychotropic medication at the time of the review. The most prescribed class of psychotropic was benzodiazepines; > 30% (*n* = 91/297) of patients in the entire cohort were already prescribed medications from this class at the time of assessment (Supporting Information S1: Appendix B).

### 3.4 Referral Details

Over a quarter of the patients (25.9%, *n* = 60/232) referred to the psycho‐oncology team had ≥ 2 reasons for referral. Anxiety was the most common reason, reported in > 30% (*n* = 75/232) of the patients, followed by depressive symptoms (19.4%, *n* = 45/232), irritability (15.1%, *n* = 35/232), and emotional lability (14.2%, *n* = 33/232). Psychotic symptoms and elated mood were indications for referral in 4.7% and 3.4% of the patients, respectively. Over half of the referrals (52.6%, *n* = 153/291) were received from medical oncology teams, followed by haematology (20.6%, *n* = 60/291) and radiation oncology (19.9%, *n* = 58/291) teams. Inpatients comprised 57.9% of the referred patients (*n* = 168/290), with the remainder being outpatients (Supporting Information S1: Appendix B).

### 3.5 Psychiatric Assessment

**FIGURE 1:**
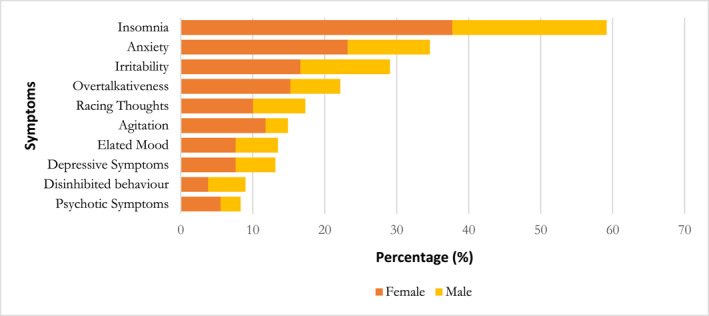
Distribution of symptoms in the cohort.

Figure 1 shows the cumulative frequency of symptoms/signs identified across the cohort after the psychiatric assessment. Insomnia was the most prevalent symptom, observed in 58.1% (*n* = 168/289) of the patients assessed.

Furthermore, 81% (*n* = 227/289) of the patients had ≥ 1 symptom on psychiatric assessment and 18% (*n* = 50/289) had ≥ 4 symptoms. The most common combination of symptoms included insomnia with either anxiety, irritability, overtalkativeness, or having racing thoughts (Supporting Information S1: Appendix B). Nine females and five males presented with all three symptoms of insomnia, anxiety, and irritability on psychiatric assessment. Additionally, four females presented with symptoms of disinhibited behaviour, elated mood, and overtalkativeness.

### 3.6 Treatment and Clinical Response

**FIGURE 2:**
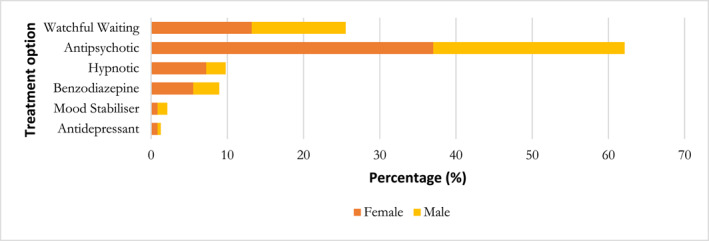
Distribution of treatment options in the study sample (*n* = 235).

Data regarding the treatments received were collected from 235 patients. A watchful waiting approach was used in 25.5% of the patients (*n* = 60/235), and psychotropics were prescribed to 74% of patients (*n* = 174/235) (Figure 2). The breakdown of the specific psychotropics used within each medication class is provided in Supporting Information S1: Appendix B. Twenty‐five patients were prescribed two concurrent psychotropics.

Data regarding patients' clinical responses at the first review by the psycho‐oncology team were available for 205 patients. In total, 90.2% (185/205) of the patients experienced either complete or partial resolution of symptoms at their first clinical review.

Among the 45 patients assigned to the watchful waiting group, 10 (22.2%) achieved complete resolution, 31 (68.9%) achieved partial resolution, and 4 (8.9%) showed no change in their symptoms. Conversely, of the 160 patients who underwent treatment with psychotropics, 14 (8.8%) achieved complete resolution, 129 (80.6%) experienced partial resolution, and 17 (10.6%) did not respond to treatment.

This aligns with our clinical practice, where milder presentations are more likely to be managed conservatively with observation, while more severe presentations, such as psychosis, are treated with psychotropic medications to ensure symptom control and patient safety. The absence of baseline severity data in our study limits the ability to make robust comparisons between treatment groups. It is important to note that the watchful waiting approach is typically reserved for less severe cases, reflecting ethical and clinical considerations that preclude leaving severe symptoms untreated.

## 4 Discussion

This study's results highlight the significant burden of symptoms associated with steroid‐induced mental disorders in patients with cancer. Our patient cohort experienced a wide variety of symptoms, with insomnia, anxiety, and irritability being the most common, and psychotic symptoms being less prevalent. These findings are consistent with those of previous studies and further suggests that the scope of steroid‐induced mental disorders extends beyond psychosis.

### 4.1 Potential Risk Factors and Symptom Profiles

The demographic data collected in this study provide valuable insights into the patient population experiencing steroid‐induced mental disorders in an oncology setting. Our cohort had a higher proportion of females than men, and the mean age of females who were diagnosed with a steroid‐induced mental disorder was statistically significantly lower. Similar findings regarding sex and age as risk factors for steroid‐induced mental disorders have been reported in previous studies within a larger population [5, 25].

Breast cancer was the most frequent malignancy among females, whereas haematological cancer was the most frequent among males and the second most frequent among females. This finding aligns with the high prevalence of breast cancer among females in Ireland, accounting for over one‐third of all cancer cases [26]. The high incidence of haematologic cancers in this cohort is notable, given the higher corticosteroid doses used in their chemotherapy compared to chemotherapy for solid tumours [27]. Given that steroid dosage is a prime predictor of steroid‐induced mental disorders, the higher prevalence of these conditions in the cohort likely reflects the effects of intensive steroid‐based treatments for haematologic cancers. Building on this, the relationship between steroid dosage and the onset of neuropsychiatric symptoms is well‐documented, with dose serving as a key determinant of risk. The Early Boston study, for instance, identified psychiatric symptoms in 1.3% of patients receiving prednisone at a dose less than 40 mg/day, 4.6% in those on 40–80 mg/day, and 18.6% in those on doses exceeding 80 mg/day [28]. In contrast, our cohort exhibited significant neuropsychiatric effects at a median daily prednisolone equivalent dose of 26.6 mg/day, far below the thresholds highlighted in the Boston study. This finding suggests that even moderate steroid doses may precipitate psychiatric symptoms, particularly in patients with cancer, where cumulative exposure and the interactions with cancer therapies may amplify vulnerability.

Chemotherapy was the predominant cancer treatment used in this cohort. The onset of sleep disturbances among patients with cancer receiving chemotherapy is well documented, with evidence indicating that these symptoms can start even before treatment initiation [29, 30]. One study also found a statistically significant reduction in sleep latency, duration, and efficiency among patients receiving high‐dose glucocorticoids in combination with chemotherapy compared with those receiving chemotherapy alone, highlighting the effect of systemic glucocorticoids on sleep [31]. In our cohort, insomnia was the most frequently reported symptom. The causes of insomnia in this population are complex and multifactorial. Additionally, a substantial proportion of the study population had oncological brain involvement. Corticosteroids are commonly used and have positive effects in patients with brain malignancies. However, distinguishing between a true steroid‐induced mental disorder and manifestations of gliomas, cerebral irradiation, or changing intracranial pressure is challenging in clinical practice [32].

Cardiovascular and endocrine diseases were the most common medical comorbidities. Depression was the most common psychiatric comorbidity. Further research is needed to ascertain whether certain comorbidities increase the risk of developing steroid‐induced mental disorders. For instance, a study of patients with multiple sclerosis revealed that a personal or family history of depression in patients who were started on high‐dose corticosteroids led to a statistically significant increase in the risk of developing (hypo)manic symptoms [33].

Most patients in this cohort were prescribed dexamethasone, and a smaller proportion was prescribed prednisolone. The high prevalence of dexamethasone use in this study is consistent with the current Health Service Executive (HSE) guidelines for counteracting nausea and vomiting induced by cancer treatment regimens [34]. The median weekly prednisolone equivalent dose was significantly higher in the patients who received prednisolone than in those who received dexamethasone. However, the median duration of steroid exposure before the onset of symptoms of steroid‐induced mental disorders was similar in both groups. The reasons for this are likely multifactorial, and the relatively small sample size makes it difficult to draw conclusions; however, a previous literature review by Stuart et al. highlighted that dexamethasone was more strongly associated with severe adverse psychological effects than prednisolone at equal doses in children and adolescents [35].

Anxiety was the most common reason for referral among the cohort. Several aspects of oncological disease and treatment can exacerbate or present as anxiety [36]. A large‐scale study of adult outpatients at a tertiary cancer centre found that 34% of patients experienced clinically significant anxiety symptoms [37]. Benzodiazepines were prescribed to 31.7% of the females and 29% of the males in this study. Furthermore, a study by Traeger et al. revealed that benzodiazepines were frequently prescribed to patients with cancer for anxiety as well as for nausea and insomnia [36]. The start dates of benzodiazepine therapy were not recorded in our cohort; therefore, it is unclear whether benzodiazepine therapy was most often initiated by the relevant medical/surgical team at the onset of the suspected steroid‐induced mental disorder or whether they were longstanding prescriptions.

Furthermore, 60% of our cohort experienced insomnia, consistent with the results of previous studies that have identified insomnia as a common neuropsychiatric side effect of steroid treatment [7, 38, 39, 40, 41].

### 4.2 Treatment Strategies

Psychotropics were initiated by the psycho‐oncology team in 74% of patients (*n* = 174/235), with antipsychotics being the most commonly prescribed drugs (Supporting Information S1: Appendix B). Atypical/SGAs were the most frequently prescribed antipsychotics. This finding is in line with a recent review on the pharmacological management of steroid‐induced psychosis, not specific to patients with cancer, which concluded that this syndrome could be effectively managed using strategies combining dose reduction or elimination of steroids with an antipsychotic medication [19].

A small but significant difference was found between the patients assigned to the watchful wait treatment strategy and those who were started on psychotropic treatment. Patients who were started on psychotropics were less likely to experience a complete resolution of their symptoms, possibly because of less severe baseline symptoms in the watchful wait group. Tapering of steroids, where possible without the addition of a psychotropic agent, is recommended in a number of studies as an effective first‐line intervention [6, 42, 43, 44]. Given the high burden of adverse effects associated with antipsychotics, further studies on tapering steroids as a primary treatment option would be beneficial.

### 4.3 Study Limitations

This study is the most extensive case series in Ireland regarding steroid‐induced mental disorders in patients with cancer, offering crucial insights into patient demographics and symptomatology to enhance oncology staff awareness. However, the study's retrospective nature and the lack of a control group limit statistical analysis. Additionally, the data were collected over a decade from a clinical database and, as a result, certain data points and variables for some patients that might have influenced the conclusions were not available. It is likely that many mild steroid‐induced psychiatric disorders are self‐limiting and may be resolved without formal recognition, introducing a potential selection bias in the study population. Additionally, the presence of pre‐existing psychiatric comorbidities in 10%–20% of patients may have influenced the likelihood of referral, representing another confounding factor. The study did not capture data differentiating between active treatment and palliative care, nor did it separate steroid use in chemotherapy regimens versus symptom management.

### 4.4 Clinical Implications

This study provides insights into the prevalence of specific symptoms and their combinations experienced by patients with cancer diagnosed with a steroid‐induced mental disorder. It serves as a call for prompt recognition of these symptoms, considering that they can have serious consequences. Our findings have the potential to support the creation of targeted screening and management strategies for patients with cancer who are at high risk of developing neuropsychiatric symptoms and will help inform future studies aimed at uncovering the underlying mechanisms and risk factors of steroid‐induced mental disorders in this patient population.

### 4.5 Conclusions

This study offers critical insights into the symptoms and management of steroid‐induced mental disorders in patients with cancer. It provides preliminary information about demographic and clinical factors associated with referral to a liaison psychiatry service for steroid‐induced mental disorders. It underscores the substantial symptom burden and highlights the need for enhanced awareness and intervention strategies. The most common symptoms were insomnia, anxiety, and irritability. The condition had a higher prevalence among females and individuals with prior depression and other comorbidities. Our results emphasise the importance of vigilant monitoring of patients receiving any steroid regimen, regardless of perceived dose‐related risks. Our study also highlights the common use of pharmacotherapy in managing these symptoms, and the need for further research to assess the effectiveness and safety of such treatments, as well as unravelling the precise aetiology and risk factors for this complex condition.

## Conflicts of Interest

The authors declare no conflicts of interest.

## Supporting information

Supporting Information S1

## Data Availability

The data that support the findings of this study are available from the corresponding author upon reasonable request.
