# Supplementary material for: Steroid‐Induced Mental Disorders in Oncology Patients: A 10‐Year Retrospective Case Series Review
Source: Psychooncology. 2025 Apr 19;34(4):e70137. doi: 10.1002/pon.70137 (PMC12009012; doi:10.1002/pon.70137)
Supplement: Supplementary file 1 — Supporting Information S1 [file PON-34-e70137-s001.docx]

# Appendix A: Data Collection Sheet

| Case ID: |  |
| --- | --- |
| Date of data collection: |  |
|  |  |
| **1. Demographics** | |
| DOB: | Continuous - interval - parametric |
| Gender: | 0 Male / 1 Female |
| Marital status: | 0 Single / 1 Married / 2 Separated / 3 Widowed |
| Highest education level attained: | 0 No formal  1 Primary  2 Lower Secondary  3 Upper secondary  4 Third level non-degree  5 Third level degree and higher |
| Age completed education: | Continuous - interval |
| Employment status: | 0 Employed / 1 Unemployed / 2 Retired / 3 self employed / 4 student / 5 Homemaker |
|  |  |
| **2. Clinical Details** | |
| Cancer Types: | \| 0 Breast \| \| --- \| \| 1 Haematological \| \| 2 Lung & thoracic \| \| 3 Gastrointestinal \| \| 4 Genital \| \| 5 Central nervous system \| \| 6 Head and neck \| \| 7 Skin \| \| 8 Urinary tract \| \| 9 Mesothelial and soft tissue \| \| 10 Endocrine \| \| 11 Bone and cartilage \| \| 12 Unknown primary \| \| 13 Multiple primary \| |
| Exact Cancer Diagnosis: | (STRING) |
| Staging: | 0 Localised / 1 local spread / 2 distant metastasis / 3 not relevant / 99 not known/not documented |
| Brain involvement: | 1 Yes / 0 No / 99 Not documented |
| First cancer diagnosis: | 1 Yes / 0 No / 99 Not documented |
| Time since cancer diagnosis (months): |  |
| Is this is a recurrence: | 1 Yes / 0 No |
| Treatment received when assessed: | 0 Chemotherapy  1 Radiotherapy  2 Combined Chemo/radiotherapy  3 Pre/post-transplant  4 Pre/post-surgical  5 None  6 Others ______________________________________ |
| Referral by: | 0 Medical Oncology  1 Radiation-oncology  2 Haematology  3 Medical  4 Surgical  5 Others: ___________________________ |
| Source of referral: | 0 Inpatient / 1 Outpatient |
| **3. Steroid Exposure (On assessment)** | |
| Previous known steroid-induced psychiatric disorder | 1 Yes / 0 No / 99 unclear |
| Type of steroids | 0 Dexamethasone  1 Prednisolone  2 Hydrocortisone  3 Prednisone  4 Others: ________________________ |
| Steroids: | 0 Pulse / 1 continuous |
| Route of administration: | 0 Oral / 1 Intravenous / 2 Mixed / 3 others |
| Dose and frequency: | __________mg/day __________ day/week _______weekly |
| Duration of steroids exposure (days): | Continuous - interval – non-parametric |
| Cumulative weekly dose of prednisolone equivalent: | Continuous - interval – non-parametric |
| Time to symptoms development: | _________ days / unclear (leave blank) |
| Date of 1^st^ psycho-oncology assessment: | date |
|  |  |
| **4. Clinical Symptoms** | |
| Reason for referral: | \| 0 Insomnia \| \| --- \| \| 1 Difficulties coping with the diagnosis \| \| 2 Elated mood \| \| 3 Agitated \| \| 4 Irritable \| \| 5 Disinhibited behaviour \| \| 6 Overtalkative \| \| 7 Change in personality \| \| 8 Psychotic symptoms \| \| 9 Depressive symtpoms \| \| 10 PDW / TSH / SI / Self-harm \| \| 11 Delirium like symptoms \| \| 12 Others ___________________________________ \| |
| Psychiatric signs/symptoms on assessment: | \| 0 Insomnia 15 Others \|  \| \| --- \| --- \| \| 1 Elated mood \|  \| \| 2 Agitated \|  \| \| 3 Irritable \|  \| \| 4 Disinhibited behaviour \|  \| \| 5 Overtalkative \|  \| \| 6 Change in personality \|  \| \| 7 Psychotic symptoms – Delusions / Auditory Hallucination / Visual Hallucination \|  \| \| 8 PDW / TSH / SI / Self-harm \|  \| \| 9 Depressive symptoms \|  \| \| 10 Delirium like symptoms \|  \| \| 11 Anxiety symptoms \|  \| \| 12 Racing thought \|  \| \| 13 Aggression (verbal / physical) \|  \| \| 14 Emotional lability \|  \| |
| Diagnosis impression:  **Details:** | 0 hypomanic episode  1 manic episode  2 manic with psychotic symptoms  3 depressive disorder  4 depressive disorder with psychotic episodes  5 delirium  6 mixed affective state / dysphoric mania  7 steroid-induced mood disorder  99 Unclear |
| **5. Treatment** | |
| Intervention: | 1 Watchful wait / 2 Commence psychotropic 0 None |
| Type of psychotropic: | 0 Hypnotic / 1 antipsychotic / 2 mood stabiliser / 3 antidepressant / 4 benzodiazepine / 5 others |
| Name of psychotropic medication prescribed: | **[STRING]** |
| Dosage and frequency: | **[STRING]** |
| Duration: | 0 Targeted / 1 continuous |
| Cumulative Duration of medication: | ______ weeks / unclear (leave blank) |
| Time to first review: | ______ day / unclear (leave blank) |
| Clinical response on 1^st^ review: | 0 No response / 1 Partial response / 2 Complete resolution |
| Duration of clinical input: | ______ weeks / 1 once-off |
| Other intervention: | 0 Stopping antidepressant  1 Reduce steroid dose  2 Stop steroid  3 Psychoeducation / leaflet information  4 others: ___________________ |
| Date of discharge from psychonc: |  |
| Symptoms on discharge | 0 Residual symptoms / 1 Complete resolution |
| Number of reviews: |  |
| Disposition: | 0 Medical team / 1 Community Mental Health team |
|  |  |
| **6. Other clinical details** | |
| Medical co-morbidities  **Details:** | \| 0 Cardiovascular disease \|  \| \| --- \| --- \| \| 1 Respiratory disease \|  \| \| 2 Endocrine disease \|  \| \| 3 Neurological disease \|  \| \| 4 Musculoskeletal \|  \| \| 5 Others ______________________________ \|  \| |
| Psychiatric co-morbidities  **Details:** | \| 0 Depression \| \| --- \| \| 1 Anxiety disorder \| \| 2 Psychotic disorder \| \| 3 Bipolar disorder \| \| 4 Other mood disorder \| \| 5 Personality disorder \| \| 6 Substance use disorder \| \| 7 Others: _______________________________ \| |
| Current existing psychotropic medication on assessment:  **Details:** | 0 Hypnotic  1 antipsychotic  2 mood stabiliser  3 antidepressant  4 benzodiazepine  5 others |

# Appendix B: Additional Tables

**Table 1: Referrals**

|  |  |
| --- | --- |
| **Reason for referral, n/N (%)** |  |
| Anxiety | 75/232 (32.2%) |
| Depressive symptoms | 45/232 (19.4%) |
| Irritability | 35/232 (15.1%) |
| Emotional lability | 33/232 (14.2%) |
| Agitation | 15/232 (6.5%) |
| Difficulties coping | 13/232 (5.6%) |
| Distressed | 13/232 (5.6%) |
| Psychotic symptoms | 11/232 (4.7%) |
| Elated mood | 10/232 (4.3%) |
| Disinhibited behaviour | 7/232 (3%) |
| Other | 32/232 (13.8%) |
|  |  |
| **Referring Team, n/N (%)** |  |
| Medical oncology | 153/291 (52.6%) |
| Haematology | 60/291 (20.6%) |
| Radiation Oncology | 58/291 (19.9%) |
| Medical | 5/291 (1.7%) |
| Surgical | 2/291 (<1%) |
| Other | 13/291 (4.5%) |
|  |  |
| **Referring Source, n/N (%)** |  |
| Inpatient | 168/290 (57.9%) |
| Outpatient | 122/290 (42.1%) |

|  | **Overall** | **Female** | **Male** |
| --- | --- | --- | --- |
| **Symptoms on Psychiatric Assessment, n (%)** | **N=289** | **N=174** | **N=115** |
| Insomnia | 171 (59.2%) | 109 (62.6%) | 62 (53.9%) |
| Anxiety | 100 (34.6%) | 67 (38.5%) | 33 (28.7%) |
| Irritability | 84 (29.1%) | 48 (27.6%) | 36 (31.3%) |
| Overtalkative | 64 (22.1%) | 44 (25.3%) | 20 (17.4%) |
| Racing thoughts | 50 (17.3%) | 29 (16.7%) | 21 (18.3%) |
| Elated mood | 43 (14.9%) | 34 (19.5%) | 9 (7.8%) |
| Agitated | 39 (13.5%) | 22 (12.6%) | 17 (14.8%) |
| Depressive symptoms | 38 (13.1%) | 22 (12.6%) | 16 (13.9%) |
| Disinhibited behaviour | 26 (9.0%) | 11 (6.3%) | 15 (13.0%) |
| Psychotic symptoms | 24 (8.3%) | 16 (9.2%) | 8 (6.9%) |

**Table 2: Individual symptoms on psychiatric assessment**

**Table 3: Most common combinations of symptoms in females**

| **Females** | **% (n/N)** |
| --- | --- |
| **Symptom combination** |  |
| 1. Insomnia and Anxiety | 23.6% (41/174) |
| 2. Insomnia and Irritability | 18.4% (32/174) |
| 3. Insomnia and Overtalkative | 13.8% (24/174) |
| 4. Insomnia and Racing Thoughts | 11.5% (20/174) |
| 5. Overtalkative and Elated Mood | 9.2% (16/174) |
| 6. Anxiety and Irritability | 7.5% (13/174) |
| 7. Anxiety and Racing Thoughts | 7.5% (13/174) |
| 8. Anxiety and Elated Mood | 6.3% (11/174) |
| 9. Irritable and Overtalkative | 5.7% (10/174) |
| 10. Irritable and Racing Thoughts | 5.7% (10/174) |
| 11. Overtalkative and Racing Thoughts | 5.7% (10/174) |
| 12. Anxiety and Depressive Symptoms | 5.2% (9/174) |

**Table 4: Most common combinations of symptoms in males**

| **Males** | **% (n/N)** |
| --- | --- |
| **Symptom combination** |  |
| 1. Insomnia and Anxiety | 19.1% (22/115) |
| 2. Insomnia and Irritability | 17.4% (20/115) |
| 3. Insomnia and Racing Thoughts | 13.0% (15/115) |
| 4. Insomnia and Overtalkative | 7.8% (9/115) |
| 5. Anxiety and Irritability | 6.9% (8/115) |
| 6. Irritability and Racing Thoughts | 6.9% (8/115) |
| 7. Overtalkative and Agitated | 6.1% (7/115) |
| 8. Anxiety and Racing Thoughts | 5.2% (6/115) |
| 9. Anxiety and Depressive Symptoms | 5.2% (6/115) |
| 10. Irritable and Overtalkative | 5.2% (6/115) |
| 11. Irritable and Agitated | 5.2% (6/115) |
| 12. Overtalkative and Racing Thoughts | 4.3% (5/115) |

**Table 5: Specific Psychotropics commenced by the Psycho-oncology team**

|  |  |
| --- | --- |
| **Hypnotic, n/N** |  |
| zolpidem | 12/174 |
| zopiclone | 8/174 |
| promethazine | 1/174 |
|  |  |
| **Antipsychotic, n/N (%)** |  |
| olanzapine | 96/174 |
| quetiapine | 32/174 |
| aripiprazole | 11/174 |
| risperidone | 6/174 |
| haloperidol | 5/174 |
|  |  |
| **Mood stabiliser, n/N (%)** |  |
| sodium valproate | 6/174 |
| lithium carbonate | 1/174 |
|  |  |
| **Antidepressant, n/N (%)** |  |
| escitalopram | 1/174 |
| mirtazapine | 2/174 |
| trazodone | 1/174 |
|  |  |
| **Benzodiazepine, n/N (%)** |  |
| alprazolam | 10/174 |
| temazepam | 2/174 |
| lorazepam | 6/174 |
| clonazepam | 1/174 |
